# Research Gaps and Recommendations to Guide Research on Assessment, Prevention, and Treatment of Moral Injury Among Healthcare Workers

**DOI:** 10.3389/fpsyt.2022.874729

**Published:** 2022-04-15

**Authors:** Shira Maguen, Brandon J. Griffin

**Affiliations:** ^1^Department of Psychiatry and Behavioral Sciences, University of California, San Francisco, San Francisco, CA, United States; ^2^San Francisco VA Healthcare System, San Francisco, CA, United States; ^3^Department of Psychiatry, University of Arkansas for Medical Sciences, Little Rock, AR, United States; ^4^Central Arkansas VA Healthcare System, Little Rock, AR, United States

**Keywords:** moral injury, healthcare workers, mental health, prevention, assessment, treatment

## Abstract

Healthcare workers face numerous occupational stressors, including some that may challenge personal and shared morals and values. This is particularly true during disasters and crises such as the COVID-19 pandemic, which require critical decisions to be made with little time and information often under personal distress and situational constraints. Consequently, healthcare workers are at risk for moral injuries characterized by stress-related and functional impacts. Although research on the evaluation and treatment of moral injury among military veterans burgeoned in the recent decade, addressing moral injury in healthcare workers and other civilians remains an important gap. In this perspective piece, we identify research gaps and make recommendations to advance future work on assessment, prevention, and treatment of moral injury in healthcare workers. We draw on empirical studies of moral injury in veterans, limited studies of moral injury in health professionals, and our clinical experiences with healthcare workers affected by moral injury.

## Introduction

Healthcare workers (HCWs) face numerous occupational stressors. This is especially true during disasters and crises such as the COVID-19 pandemic, which require critical decisions to be made with little time and information often under personal distress and situational constraints. In doing so, HCWs may transgress their moral beliefs or expectations by what they did or failed to do, even when their behavior is consistent with recommended standards of care. For example, HCWs might perceive considerable suffering to be the result of their actions when restricting access to testing or treatment resources due to lack of supply or denying visitors to patients or care facility residents who are dying of complications related to COVID-19. They may consequently sustain a moral injury, defined as psychological, behavioral, social, and religious/spiritual problems stemming from perceived transgression of internalized moral beliefs rooted in cultural, organizational, and group-based ethical norms (1).

Studies of moral injury proliferated during the recent decade, most of which focused on military personnel and veterans exposed to potentially morally injurious events (PMIEs) in the context of combat (e.g., killing in war) (2). Our research demonstrates that post-9/11 veterans exposed to a PMIE are at risk of experiencing mental health problems, functional impairment, and suicide, particularly the 20% who report transgressing their own moral values (3, 4). Critically, HCWs' experiences of the COVID-19 pandemic illuminated the nascent construct of moral injury, uncovering a considerable gap in our understanding of moral injury among non-military populations. We identify research gaps and make eight recommendations below to advance future work on assessment, prevention, and treatment of moral injury in healthcare workers. We draw on empirical studies of moral injury in veterans, limited studies of moral injury in HCWs, and our clinical experiences with HCWs affected by moral injury.

## More Research Is Needed to Examine the Unique Impacts of Moral Injury and Risk and Protective Factors Among HCWs

Several studies demonstrate that moral injury is both potentially prevalent and associated with increased distress and decreased functioning in HCWs (5–8). Norman et al. (6) found that 53–88% of healthcare workers endorsed moral distress due to family-related (e.g., worry about the effect of COVID-19 on one's ability to care for dependents), infection-related (e.g., worry about infecting patients with COVID-19), and work-related concerns (e.g., worry about having to make difficult decisions that prioritize the health of one patient over another). Moreover, moral distress was associated with COVID-19-related posttraumatic stress disorder (PTSD) symptoms, burnout, and impaired occupational and relationship functioning. Importantly, in a longitudinal study, exposure to PMIEs was associated with suicidal ideation among some HCWs (7).

When examined over time, moral distress may linger, while other mental health symptoms dissipate. In a longitudinal study, rates of mental health symptoms declined over several months while rates of moral injury remained stable (8). Given preliminary indications of a unique trajectory that may be different from other mental health symptoms, more research is needed to distinguish the unique impact of moral injury from general mental health outcomes in HCWs. Related, it will be important to examine how moral injury is associated with functioning, given the relationship between moral injury and functioning in veterans (3); specifically, those who reported transgressions against their own morals showed trajectories that were characterized by poor or declining functioning in several domains (e.g., work and relationship functioning) (9). Similarly, Borges et al. (5) found an association between exposure to PMIEs and poorer self-reported psychosocial functioning among HCWs. If moral injury symptoms are impeding HCWs from performing optimally at work, this can lead to additional moral distress (e.g., through poor decision making due to stretched cognitive resources) and ultimately to decreased job satisfaction and burnout. If moral injury symptoms are creating strained relationships, this may lead to greater challenges at home and thus remove sources of support that could mitigate further moral injury symptoms.

An important caveat is that HCWs are a heterogenous group, and experiences with moral injury will likely vary across different individual, occupational, organizational, and cultural groups. Williamson, Murphy, and Greenberg (10) identify attributes of HCWs who may be at greater risk of moral injury including those who are exposed to death and dying as part of their job, work with vulnerable individuals (e.g., children), perceive leadership to be unsupportive, feel unprepared for the emotional/psychological consequences of decisions (e.g., less experienced providers), and those who have a history of exposure to trauma and violence. Risk beyond the individual level may extend to subgroups including medical trainees and early career providers who are likely to witness medical errors but who may be overlooked as participants in ensuring patient safety (11). Finally, given that HCWs are a resilient group, it will also be important to understand individual protective factors that mitigate against the development of moral injury-related distress.

## Better Measures of Moral Injury Are Required to Inform Care and Research

One of the biggest barriers in being able to address moral injury among HCWs is the absence of culturally appropriate, valid, and reliable measures of moral injury. Currently, the majority of moral injury measures focus on military-related moral injury, most commonly in the context of war or deployments. Additionally, within these existing measures, a few conflate exposure to PMIEs and associated moral injury symptoms (2, 12). Typically current moral injury measures capture one or the other; for some of those measuring moral injury symptoms, these symptoms are not always reliably indexed to a PMIE (but rather vaguely ask about experiences), creating room for measurement error and the likelihood of capturing overall distress rather than the symptoms of moral injury in particular. What is needed is a measure that is validated with HCWs, indexed to PMIEs specifically, and includes a variety of moral injury symptoms that manifest as a result of these exposures, including the hallmark symptoms of moral injury (e.g., guilt, disgust, inability to self-forgive, self-sabotaging behaviors). Additionally, because moral injury symptoms have been found to potentially linger, a moral injury measure that is sensitive enough to assess changes over time would help illuminate trajectories of moral injury at various phases of recovery (12).

## Extension of Moral Injury to HCWs Requires a Mixed Methods Approach

While some preliminary efforts to assess and treat moral injury have involved adapting tools developed for veterans to HCWs as a first step, we recommend a ground-up, mixed methods approach to more deeply and comprehensively understand the impact of moral injury on HCWs both during and outside of pandemics. To that end, few mixed methods studies have been published to date that focused on HCWs' experiences of moral injury. Hand in hand with measuring rates of moral injury, striving to better characterize moral injury in the voices of HCWs is a critical next step. While nomothetic studies indicate that HCWs experience moral distress, we lack a more nuanced understanding of the origins, exacerbating factors, and specific impacts. A need exists for mixed methods idiographic work to understand the unique subjective experiences of HCWs who sustain a moral injury, dynamics between frontline workers and administrators/policy makers that may contribute to moral injury, HCWs' preferences for healing, and critical aspects related to delivery of moral injury care. Notable studies from the military-related moral injury literature that may be useful guides survey veterans' reactions to killing in war (13) and providers' experiences of delivering treatment for moral injury (14).

## Construct Validity Is an Essential Part of Establishing Conceptual Clarity of Moral Injury Among HCWs

Within the literature on military service members and veterans who are often exposed to multiple and complex highly stressful events, scholars prioritized differentiation of moral injury from posttraumatic stress disorder (PTSD). Whereas PTSD is associated with exposure to events that threaten one's physical integrity and characterized by maladaptive biological and behavioral fear responses (e.g., exaggerated startle), a growing consensus suggests that moral injury is associated with exposure to events that transgress one's core beliefs and is expressed in self-deprecating, self-directed cognitions, emotions, and behaviors (e.g., guilt, powerlessness, disgust, demoralization, self-sabotaging behaviors) (1, 15). As the literature on moral injury extends to non-military populations, gaining conceptual clarity about the construct of moral injury is important to understand how it is similar and different from other related constructs. For example, burnout, conceptually different than moral injury, is a syndrome of poor work engagement characterized by emotional exhaustion, depersonalization, and lack of personal accomplishment (16). A number of editorials (17) and few pioneering empirical studies (18) have already been published connecting moral injury and burnout; however, empirical studies that investigate areas of distinction (and overlap) are needed. When moral injury co-occurs with other clinical syndromes in a given population, innovative methodologies such as networking analyses can help elucidate the complex relationships between moral and psychological distress (19).

## There Is a Need to Develop and Test Culturally Appropriate Moral Injury Interventions for HCWs

Of HCWs that experience some mental health symptoms and signs of moral injury, most will heal on their own and through the support of friends, family and coworkers. In fact, a rapid review of the research in HCWs during pandemics found that systems-level interventions, rather than individual ones, may alleviate distress for most providers without the need for specialized mental health intervention (20). Additionally, the authors recommend a stepped-care mental health response that includes “proactive health care leadership, psychotherapeutic intervention, and referral to specialized care” to most effectively allocate resources and care to those in need. They also highlight that for those that need specialized care, it is important to commit resources to develop and test evidence-based mental healthcare for HCWs following pandemics. Unfortunately to our knowledge, evidence-based psychotherapies (EBPs) addressing moral injury for HCWs do not yet exist, although there are some studies with veterans that may shed light on which types of treatments may be most helpful.

There are now several psychotherapeutic interventions for veterans with moral injury that have preliminary support and are undergoing more rigorous testing through randomized controlled trials (RCTs). These include Adaptive Disclosure (AD) (21), Impact of Killing (IOK) (22), Trauma-Informed Guilt Reduction Therapy (TrIGR) (23), Acceptance and Commitment Therapy for Moral Injury (ACT-MI) (24), Building Spiritual Strength (BSS) (25), and the Mental Health Clinician and Community Clergy Collaboration (MC3) (26). There is also some evidence that certain PTSD treatments can help with moral injury symptoms (e.g., Cognitive Processing Therapy) (27). Many of these moral injury treatments directly deal with the hallmark symptoms of moral injury, such as decreasing guilt and/or shame, and increasing self-compassion and self-forgiveness. While these treatments have all demonstrated promise with veteran and/or military populations, most have been specifically crafted to deal with moral injury related to war. Although there may be some similarities in issues that are faced by frontline HCWs during a pandemic and veterans at war, the situations that frontline HCWs experience are unique and require study and attention. Notably, as stated above, ground-up, mixed methods studies are needed to identify the ways in which HCWs are most impacted and want to be helped. For example, when it comes to treatment, it is critical to better understand needs and preferences of HCWs rather than adapt existing treatments to HCWs.

In one qualitative study of HCWs, the following suggestions were made about how to help remedy moral injury, including: (1) providing counseling or other emotional support; (2) offering peer support (whether formal or informal); (3) educational and ethical support; (4) wellness offerings; and (5) spiritual or faith-based support (28). To date, there have been some treatments that have been created to target moral injury among HCWs and have shown preliminary acceptability and feasibility. For example, an Acceptance and Commitment Therapy based online group intervention for moral injury has shown some promise (29), but has not yet been tested in a RCT. Although other preliminary treatments are in development, research published thus far is more descriptive. There are also a few online e-packages that have been developed that offer a combination of psychoeducation and self-help, but none have been formally tested yet.

## A Need Exists to Address Access and Barriers to Moral Injury Care

Notwithstanding the pioneering work of those developing, testing, and disseminating treatments for moral injury with veterans, the existing treatments have notable limitations. Chief among these limitations is that all are delivered in specialty mental health clinics by licensed independent providers and/or chaplains and require eight to twelve 60- to 90-min sessions. The existing treatments are therefore susceptible to known access barriers and would require substantial resource investment to scale beyond specialty mental health settings (30). An opportunity exists to develop higher reach, less intensive interventions to address moral injury and its sequelae for busy HCWs and within the broader community. This could include strategies that implore innovative modalities of delivering care such as web-based, self-management tools that involve synchronous digital interactions between patients and eHealth applications, as well as peer responder interventions that could leverage the infrastructure and relational capital that already exist within healthcare organizations. These modalities may also reduce stigma about seeking mental healthcare among HCWs, which is a known and consistent barrier. Additional work is also needed to explore the association between moral injury symptoms and healthcare utilization, help-seeking, etc.

## The Role of Organizations and Leadership in Prevention and Healing of Moral Injury Is Critical

In addition to studying individual factors associated with moral injury, it will be critical to include organizational factors that may exacerbate or mitigate moral injury, given that HCWs function within teams and systems of care within complex organizational structures. Among veterans, Currier et al. (31) observed that organizational (e.g., perceiving leadership to be out of touch), environmental (e.g., need for rapid decision-making), and cultural/relational circumstances (e.g., dehumanization toward consumers) all contribute to a climate in which moral injury is more likely to occur. Consequently, healthcare organizations can support HCWs and mitigate the impact of moral injury by planning for prevention at the primary (e.g., dissemination of information about moral injury, encouraging seeking informal support, proactive check-ins by leadership), secondary (reducing stigma and training staff to identify moral injury signs in peers) and tertiary levels (accessible, confidential, and rapid availability of mental health services) (10, 32).

Existing studies of HCWs consistently find that leadership support is a protective factor and is negatively associated with moral injury (28, 33). In their qualitative examination of HCWs in the pandemic, Nelson et al. (28) found that the importance of organizational infrastructure (e.g., availability of resources, clear communication) and leadership support were two consistent themes that emerged in the context of moral injury. Leadership support was associated with factors such as being heard and having concerns addressed, trustworthiness, empathy and being valued, and leaders being present or visible (27). Preliminary studies suggest that leadership could play an important role in the prevention and healing from moral injury, and that offering support to healthcare workers from those in these leadership roles cannot be underestimated. HCWs are embedded within organizational structures that are complex, with hierarchical structures, and support and normalization of some reactions to their complex work by leaders can go a long way in mitigating downstream issues. Similar to leadership support in the military increasing cohesion and decreasing mental health concerns, just so leadership among HCWs can also make an important impact for those who are caring for others' during a pandemic.

## The Intersection Between Moral Injury and Health Equity Is an Important Area of Inquiry

As Litz et al. (1) suggest, one's moral beliefs and expectations are rooted in cultural, organizational, and group-based ethical norms. We internalize “what is right” based on various social and cultural norms to develop a moral scheme, which is comprised of numerous overlapping and potentially conflicting beliefs. Moral injury often results in situations that elicit an inevitable sense of transgression; what is right by one standard (e.g., maximizing individual patient's chance of survival by providing the highest quality of care) differs from what is right by another standard (e.g., conserving scarce testing and treatment sources due to lack of supply). Acknowledging conflicting moral perspectives, accepting naturally occurring emotions, and taking action to facilitate moral repair will likely require compassion toward others and oneself from HCWs and cultural humility from the clinicians who aim to understand another's moral distress (34).

The majority of moral injury studies have drawn samples from White, educated, industrialized and individual-focused (vs. collectivist) populations. Greater representation of diverse individuals is sorely needed in this research, especially to the extent that culture shapes what moral beliefs and expectations an individual internalizes. The COVID-19 pandemic has highlighted long-existing health inequities within the United States, with infected individuals who identify with underprivileged groups, such as Black, indigenous, and people of color (BIPOC) having higher rates of infection and lower rates of survival (35). The pandemic has also highlighted long-existing systemic inequities and racist structures of care that providers must work within (35). Providers may feel complicit in the discriminatory impact of COVID-19 on underprivileged communities, and thus experience moral distress. Concurrently, the pandemic has also created the opportunities to have challenging conversations that can start to recognize and undo these harmful structures (36), providing HCWs with advocacy opportunities that may serve as an outlet to resolve moral distress. But the other side of the coin is that many may take on more personally to try to ameliorate these systemic issues, and ultimately experience burnout.

## Conclusion

Given the tremendous number of stressors that healthcare workers have had to endure during their careers, and during the COVID-19 pandemic, it is important to best understand how to assess, prevent and treat the ripple effects of these stressors, including the impact of moral injury. First and foremost, it is critical to acknowledge the tremendous resilience and growth of HCWs as they have navigated a pandemic and provided the world with so much comfort and healing. Related, it is important to acknowledge that although we have focused on moral injury, which impacts emotional, psychological, behavioral, and spiritual wellbeing, moral distress occurs on a continuum and does not always cause lasting distress [please see Riedel et al. (37) for a recent scoping review of moral distress and moral injury in HCWs]. To guide future research in this area, we suggest eight recommendations, highlighting that critically, this research needs to be culturally attuned and directly involve the input and diverse voices of healthcare workers on the frontlines. More mixed methods and longitudinal research is needed to characterize moral injury in HCWs, understand systemic and individual factors that contribute to moral injury, and better understand treatment needs, particularly of those who experience moral injury and may not be easily identified. More honed measurement of moral injury can help guide each of these tasks as well as capture improvement over time. It is also critical to understand how the diverse workforce of HCWs have experienced health inequities and other systemic harms that contribute to moral injury. Overall, better understanding how to characterize and heal from moral injury will require cultural humility, compassion, and attunement as we support our HCWs in their restorative journey.

## Author Contributions

SM and BG conceptualized, outlined, wrote, and edited the current perspectives article. All authors contributed to the article and approved the submitted version.

## Conflict of Interest

The authors declare that the research was conducted in the absence of any commercial or financial relationships that could be construed as a potential conflict of interest.

## Publisher's Note

All claims expressed in this article are solely those of the authors and do not necessarily represent those of their affiliated organizations, or those of the publisher, the editors and the reviewers. Any product that may be evaluated in this article, or claim that may be made by its manufacturer, is not guaranteed or endorsed by the publisher.
